# Phasor-Based Endogenous NAD(P)H Fluorescence Lifetime Imaging Unravels Specific Enzymatic Activity of Neutrophil Granulocytes Preceding NETosis

**DOI:** 10.3390/ijms19041018

**Published:** 2018-03-29

**Authors:** Ruth Leben, Lennard Ostendorf, Sofie van Koppen, Asylkhan Rakhymzhan, Anja E. Hauser, Helena Radbruch, Raluca A. Niesner

**Affiliations:** 1 Biophysical Analytics, German Rheumatism Research Center, 10117 Berlin, Germany; ruth.leben@drfz.de (R.L.); vakoppen.s@gmail.com (S.v.K.); asylkhan.rakhymzhan@drfz.de (A.R.); 2 Neuropathology, Charité—Universitätsmedizin, 10117 Berlin, Germany; lennard.ostendorf@charite.de (L.O.); helena.radbruch@charite.de (H.R.); 3 Intravital Microscopy and Immune Dynamics, Charité—Universitätsmedizin, 10117 Berlin, Germany; hauser@drfz.de; 4 Immune Dynamics, German Rheumatism Research Center, 10117 Berlin, Germany

**Keywords:** NAD(P)H (nicotinamide adenine dinucleotide (phosphate)), fluorescence lifetime imaging, neutrophil granulocytes, NADPH oxidase, NETosis

## Abstract

Time-correlated single-photon counting combined with multi-photon laser scanning microscopy has proven to be a versatile tool to perform fluorescence lifetime imaging in biological samples and, thus, shed light on cellular functions, both in vitro and in vivo. Here, by means of phasor-analyzed endogenous NAD(P)H (nicotinamide adenine dinucleotide (phosphate)) fluorescence lifetime imaging, we visualize the shift in the cellular metabolism of healthy human neutrophil granulocytes during phagocytosis of *Staphylococcus aureus* pHrodo™ beads. We correlate this with the process of NETosis, i.e., trapping of pathogens by DNA networks. Hence, we are able to directly show the dynamics of NADPH oxidase activation and its requirement in triggering NETosis in contrast to other pathways of cell death and to decipher the dedicated spatio-temporal sequence between NADPH oxidase activation, nuclear membrane disintegration and DNA network formation. The endogenous FLIM approach presented here uniquely meets the increasing need in the field of immunology to monitor cellular metabolism as a basic mechanism of cellular and tissue functions.

## 1. Introduction

Since 1990, when Winfried Denk et al. first introduced the concept of two-photon laser-scanning microscopy and showed the unique benefits of ultra-short pulsed, near-infrared excitation for imaging highly-scattering biological tissue [1], this laser-based technology tremendously expanded its field of application, especially in neurosciences [2,3,4] and immunology [5] but also in other disciplines such as nephrology [6] or developmental biology. Over almost three decades of multi-photon microscopy research, we have gained unprecedented insights into cellular dynamics and communication in living organisms [7,8,9,10,11,12]. In the past years, the further development in multi-photon microscopy heads towards a better understanding of function in living cells, tissue or even organisms. In this sense, fluorescence lifetime imaging microscopy (FLIM) is a promising technology, which probes the immediate microenvironment of molecules, as the basis of cellular function [13,14,15,16,17,18,19]. FLIM generates images in which contrast is obtained by the excited-state lifetime τ of fluorescent molecules instead of their intensity, thus, having negligible experimental bias. Fluorescence lifetimes are typically in the range of a few nanoseconds, and are sensitive to ion concentrations, pH, viscosity and other cellular or extracellular parameters [20,21,22,23]. Various technologies have been developed to perform FLIM, both in frequency-domain and in time-domain. The time-correlated single-photon counting (TCSPC), which requires pulsed excitation as delivered by multi-photon microcopy, proved to be a highly versatile (but rather slow, i.e., typically 1–10 s/frame) technology to comprehensively acquire the molecular complexity within biological samples. TCSPC directly measures the fluorescence decay of all contained fluorophores, however, its thorough analysis remains a challenge [24,25].

A special application of FLIM is based on probing the endogenous fluorescence of the ubiquitous co-enzymes nicotinamide adenine dinucleotide (NADH) and nicotinamide adenine dinucleotide phosphate (NADPH), hereafter NAD(P)H. The fluorescence lifetime of the free NAD(P)H is much shorter than that of enzyme-bound NAD(P)H [22]. This allows us to distinguish between metabolically inactive, e.g., dormant or dying cells on the one hand and highly vital cells on the other. The fluorescence lifetime of the enzyme-bound NAD(P)H is strongly dependent on the NAD(P)H binding-site on the enzyme as measured under extra-cellular conditions in mixtures of NAD(P)H, various enzymes and corresponding substrates [26,27,28]. These differences are related both to the enzymes as well as to the coenzyme itself, as reported by Blacker et al. [29].

The members of the NADPH oxidase (NOX) family, i.e., NOX1 through 4 and DUOX1 and 2, are membrane-bound enzymes specifically expressed in different cell subsets [30]. They fulfill in the first line a pathogen-clearing function based on oxidative burst generation, next to signaling functions triggered by intracellular reactive oxygen species (ROS). Using NAD(P)H-FLIM, we have shown that a preferential activation of NOX enzymes against basic, survival enzymes leads to a specific increase in the fluorescence lifetime of enzyme-bound NAD(P)H, independent of cell type or NOX isotype [4,26,31].

Polymorphonuclear cells (PMNs) are cells of the innate immune system mainly engaged in clearing fungi and bacteria. After pathogen engulfment, the cells activate NADPH oxidases (mainly NOX2) to catalyze massive ROS production and, thus, to specifically degrade the pathogens, either within phagosomes or outside of the cells. If the pathogens are too large [32] or too numerous [33], neutrophils are able to enter a specific cell death program called NETosis [34]. In ROS are an essential part of the canonical NETosis pathway. Consequently, neutrophils of chronic granulomatous disease (CGD) patients, carrying mutations in the NOX system, are severely compromised in their ability to produce NETs [35]. In recent years, rapid/early NET generation without NETosis (sometimes named *vital NETosis*) has been demonstrated, that is under certain circumstances supposed to be ROS-independent [36]. However, different studies with various stimuli leading to NETosis show contradictory results in response to the same stimuli regarding this process. Therefore, we aimed to develop a reproducible unbiased and effective approach for quantitative analysis of NOX-dependent NETosis.

Here, we employ dynamic NAD(P)H-FLIM to study changes in the metabolic activity of human polymorphonuclear cells during pathogen-induced phagocytosis and found typical enzymatic finger-prints, which distinguish suicidal NETosis from other pathways of cell death. Our multi-photon TCSPC-based fluorescence lifetime imaging method in combination with the phasor-approach for data evaluation represents a generally valid, highly reliable technology, allowing the investigation of cellular metabolism as an underlying mechanism of life. Our approach takes advantage of the newest hardware developments allowing for optimal photon yield combined with self-written, model-free algorithms. Hence, with our approach we meet the increasing demand to monitor cellular metabolism at subcellular resolution, which gains attention especially in the field of immunology.

## 2. Results

### 2.1. Endogenous NAD(P)H Fluorescence Lifetime Imaging Unravels the Metabolism of Innate Immune Cells on an Enzymatic Basis

**Figure 1:**
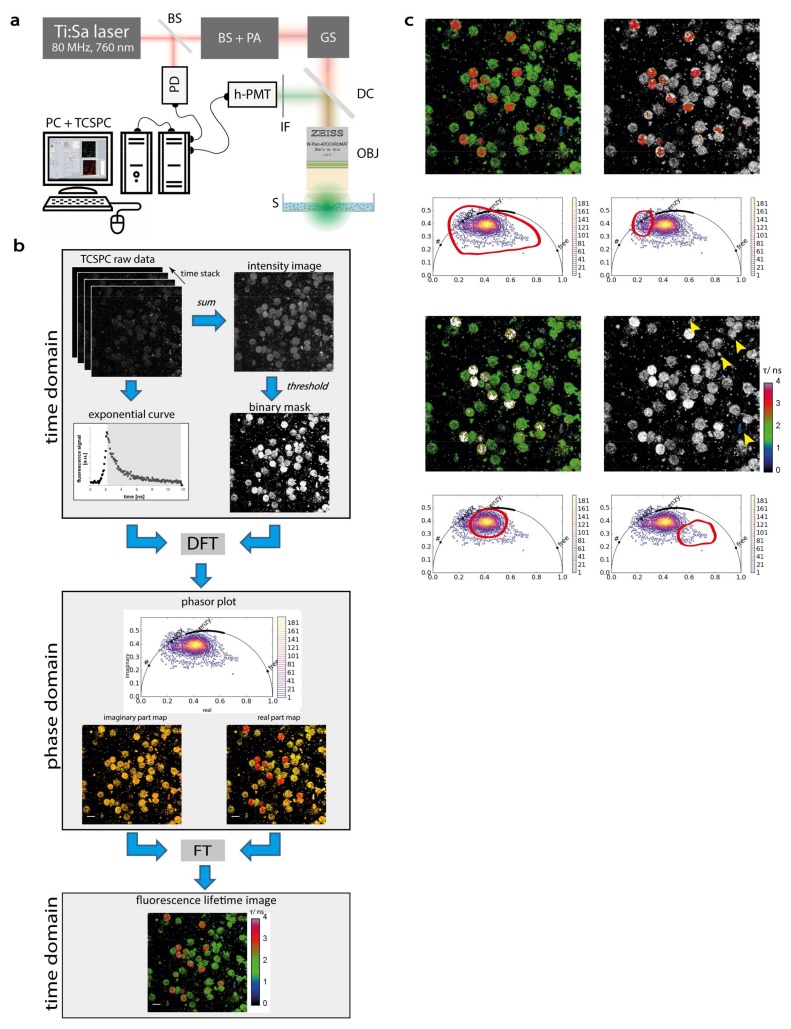
Acquisition and evaluation of NAD(P)H fluorescence lifetime imaging data in cells (**a**) Experimental setup. The beam of a 80MHz pulsed titanium sapphire laser (Ti:Sa) at 760 nm passes a beam shaper (BS), including a telescope and pulse compressor, and a λ/2 plate-based power attenuator (PA). A galvoscanner (GS) scans the beam over the sample (S). The laser beam is focused by a 20× water immersion NA 1.05 objective (OBJ). Excitation and emission light are separated by a dichroic mirror (DC, 695 nm), the emission light passes an interference filter (IF 466/40) and is time-resolved detected by a time-correlated single photon counter (TCSPC) equipped with a Hybrid-PMT (h-PMT). A beam splitter (BS) reflects 5-10% of the laser light onto a photodiode (PD) to time-synchronize the TCSPC. (**b**) Data acquisition and evaluation. The TCSPC raw data containing 227 single images form in a time-domain plot the typical exponential curve. A binary mask to exclude background pixels is created by thresholding the sum image of the single 227 images of the decay curve. Each pixel in the raw data set is converted by discrete Fourier Transformation to the phase domain (with 80MHz modulation frequency); each pixel has now a real and an imaginary part, which can be shown as real and imaginary part maps and give the coordinates of the phasor plot respectively. The phasors of free NAD(P)H, NAD(P)H bound to metabolic, survival enzymes and NADPH bound to NADPH oxidase are marked on the normalized half circle from shorter (right) to longer (left) lifetimes. In order to re-calculate a fluorescence lifetime image, the phasor data need to be back-transformed to time domain by continuous Fourier transform. (**c**) Back grating of data points in phasor plot to image pixels. In the phasor plot freehand selected data points (red line) are colored represented in the lifetime image in contrast to the unselected pixel shown in grayscale, to visualize the correspondence between populations in the phasor plot to those in the lifetime images. The data points reaching toward “free NAD(P)” are blue in the lifetime image and are marked by arrow, due to bad contrast from blue to black. Scale bar is 20 µm. In the phasor plots in b and c “free” encodes free NAD(P)H, “enzym.” NAD(P)H bound to metabolic enzymes, “NOX” NADPH bound to NADPH oxidases and “#” oxidized lipids as defined by Datta et al. [37].

The ubiquitous coenzymes NADH and NADPH, hereafter NAD(P)H, are key players of the basic metabolism as well as of various other functions in cells. At the same time, they are endogenous fluorescence probes, since they can be selectively detected by two-photon microscopy when they are excited at 760 nm and their fluorescence is detected at 460 nm (Figure 1b). The fluorescence decay of the coenzymes contains a short-lived component, i.e., the fluorescence decay of free NAD(P)H (τ_1_ ~ 450 ps) and a longer-lived component, i.e., the fluorescence decay of enzyme-bound NAD(P)H (τ_2_). Enzyme-bound NAD(P)H is involved in various biochemical reactions within the cell, catalyzed by the enzymes to which these co-enzymes are bound (Figure 1b). The short fluorescence lifetime is the average over all conformational structures of NAD(P)H, spanning between approx. 200 and 700 ps.

**Figure 2:**
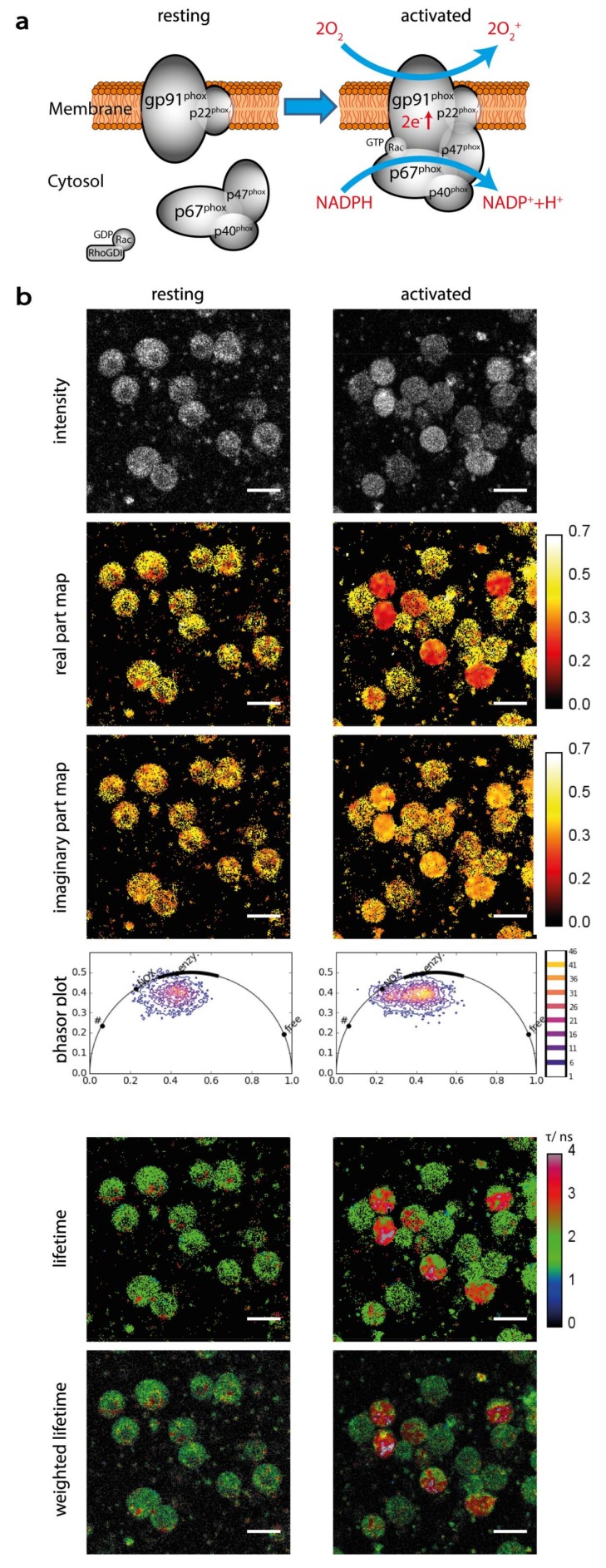
Selective detection of NAD(P)H-dependent enzymatic activity (**a**) Resting and active NADPH oxidase 2 (NOX2). The subunits gp91^phox^ and p22^phox^ form the membrane-associated component of the enzyme, whereas p67phox, p47phox and p40phox the cytosolic cyclic hetero-trimer. If stimuli, e.g., pathogens, are present, the cytosolic part translocates to the membrane-associated part under phosphorylation of p67 and the activable enzyme assembles. The assembled NOX2 is now able to bind NADPH and to catalyze the oxidation of O_2_ to the highly reactive O_2_^−^. This is rapidly transformed in chain reaction to various reactive oxygen species (ROS). (**b**) Validation of the FLIM approach on resting and PMA-stimulated monocytes, respectively. The pixel values in the real and imaginary part maps reach due to normalization from 0 to 1 and from 0 to 0.5, respectively. The calibration bar was set to 0 to 0.7 for better visibility. The images in the sixth row show fluorescence lifetime images weighted by the intensity (sum) image. Green color in the lifetime images indicates the resting status; red activated cells, in which NADPH binds to NOX2. These two populations can also be identified in the phasor plots, since the activated cells are displayed at the position marked as NADPH oxidase, not present in resting cells. Scale bar = 20 µm. In the phasor plots in b and c “free” encodes free NAD(P)H, “enzym.” NAD(P)H bound to metabolic enzymes, “NOX” NADPH bound to NADPH oxidases and “#” oxidized lipids as defined by Datta et al.

By transferring the fluorescence decay of cellular NAD(P)H to the frequency domain using the phasor approach to FLIM, we characterized the enzymatic activity of innate immune cells, i.e., polymorphonuclear cells and CD11b^+^ monocytes (Figure 1b). Cells with generally low metabolic activity display a high concentration of free NAD(P)H and are located within the half-circle near to values between 400 and 450 ps, characteristic for free NAD(P)H. We previously found that cells treated with KCN or NaCN, i.e., dying cells mainly containing free NAD(P)H [38], display short fluorescence lifetimes of approx. 450 ps [26,31]. Moreover, cells kept on ice, at 4 °C showed a similar fluorescence lifetime behavior [13]. Metabolically active cells, are located next to values between 1500 and 2500 ps in the phasor plot. If the cells are stimulated to activate specific enzymes, such as phorbol-myristate-acetate (PMA) mediated stimulation of the NADPH oxidase family in monocytes (Figure 2b), the signal in the phasor plot shifts towards 3600 ps.

Since the NADPH oxidase NOX2—the main catalyzer of oxidative burst and highly expressed in PMNs-plays a central role in the process of phagocytosis, we focus on the detection of its activation during phagocytosis of *S. aureus* coated beads by means of NAD(P)H-FLIM.

### 2.2. NADPH Oxidase Activation Co-Localizes with Phagocytosed Staphylococcus aureus Beads

Co-cultures of PMNs isolated from human blood with *S. aureus*-coated *pHrodo* beads were imaged over up to 80 min by conventional two-photon fluorescence microscopy and NAD(P)H-FLIM. Thereby, engulfment of the beads, the corresponding change in pH value and the activation of NOX2 catalyzing the oxidative burst were correlatively monitored (*Movie 1*).

**Figure 3:**
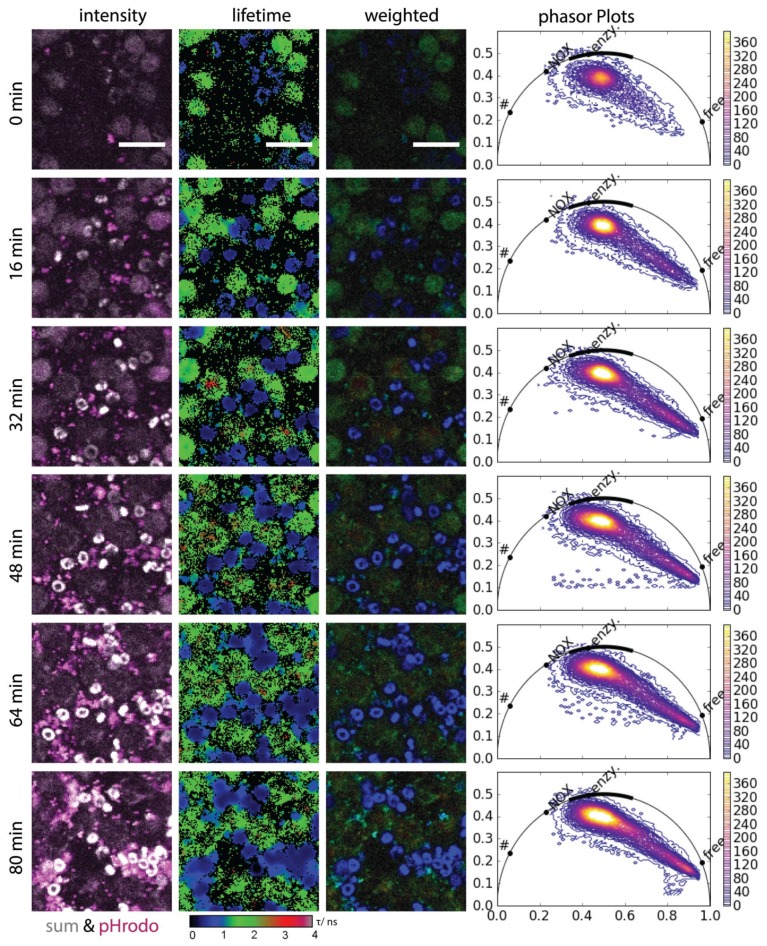
NAD(P)H-metabolism during phagocytosis. Selected frames of time-lapse NAD(P)H-FLIM data of healthy human neutrophil granulocytes phagocytosing *Staphylococcus aureus* pHrodo™ beads (representative for *n* = 150 cells per individual, two healthy individuals). The first row shows the merged images of NAD(P)H fluorescence (grey) and acidic pHrodo™ beads (magenta). The second and third rows show corresponding original and intensity-weighted fluorescence lifetime images. During the imaging time, the beads are taken up by the neutrophils and became fluorescent due to the pH shift within the phagosomes. Both the color map of FLIM images and the phasor plots show over time a shift to activated NADPH oxidase 2 followed by a shift towards free NAD(P)H, which indicates cellular death. For a better visibility, only a 124 × 124 pixel area is shown from the original data (Movie 1). The phasor plots rely on the total area of 512 × 512 pixels, the movie is shown in original dimensions. In the movie, the ROI showed in the figure is marked. Scale bar = 20 µm. In the phasor plots in b and c “free” encodes free NAD(P)H, “enzym.” NAD(P)H bound to metabolic enzymes, “NOX” NADPH bound to NADPH oxidases and “#” oxidized lipids as defined by Datta et al.

Using a feed-back loop between the fluorescence lifetime images and the phasor plot, we show that PMNs freshly mixed with *pHrodo* beads and kept at 37 °C have a high basic enzymatic activity, typical for their survival function. After the beads are engulfed by PMNs, the pH value in their microenvironment decreases a process concurrent with the appearance of red fluorescence of the beads. The pHrodo beads alone are non-fluorescent in medium (Figure S1). At the sites where acidic pHrodo beads are present, the NAD(P)H fluorescence lifetime in PMNs shifts towards longer values, i.e., 3600 ps. Hence, the basic enzymatic activity at these sites shifts towards a preferential enzymatic activity of catalyzers of oxidative burst—mainly NOX2 in PMNs (Figure 3). In blood-derived PMNs from two healthy individuals (*n* = 150 cells per individual), we measured a significant increase of the real component in the phasor plots in the range 0.1–0.5 at the initial time point and 80 min after adding *S. aureus* pHrodo beads, respectively, i.e., *p* = 0.02 using Student *t*-test. Interestingly, pHrhodo *E. coli* beads have been used only recently to correlate NETosis and phagocytosis [39].

**Figure 4:**
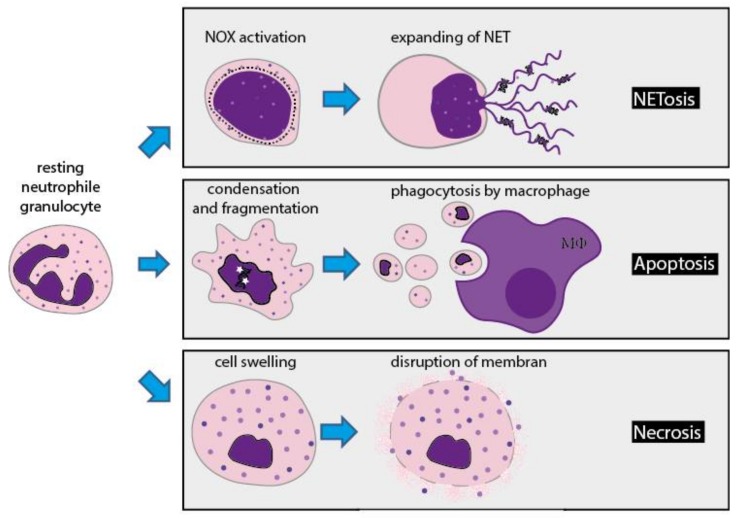
NETosis vs. apoptosis/necrosis. Three different pathways of cellular death of neutrophil granulocytes. (**a**) NETosis is the beneficial suicide. If the cells are outnumbered by pathogens, the cell nuclei round up and almost fill the cell while the nuclear membrane disintegrates and the nuclear content merges with the content of the granules. The cell contracts, DNA and granular content leak out the cells and forms NET. NET immobilizes pathogens extracellularly, making them an easy target for other immune cells. (**b**) Apoptosis is the programmed (natural) cell death. The cell shrinks, the chromatin condenses and the cell membrane blebs. The cell disintegrates to fragments, which are subsequently phagocytosed e.g., by macrophages (MΦ). (**c**) Necrosis is the traumatic cell death. The cell swells up due to acute cellular injury, until the cell membrane disrupts and releases all cellular contains uncontrolled into the extracellular space. Both NETosis and necrosis leads, in contrast to apoptosis, to tissue inflammation.

After approx. 40 min, the NAD(P)H fluorescence lifetime in several PMNs significantly shifts towards 450 ps, towards low enzymatic activity, i.e., a decrease in the real component of the phasor plot in the range 0.6 to 1.0, *p* = 0.01 by Student *t*-test. Since this behavior is similar to that of PMNs as well as other cells after treatment with NaCN, we attributed it to cell death (Figure 3). In this context, we question whether the cell death following NOX2 activation is associated with NETosis, as a PMN-typical clearing mechanism, or whether it is a general mechanism preceding any pathway leading to cell death (Figure 4).

### 2.3. NADPH Oxidase Activation Precedes NETosis but Not Other Pathways of Cell Death

**Figure 5:**
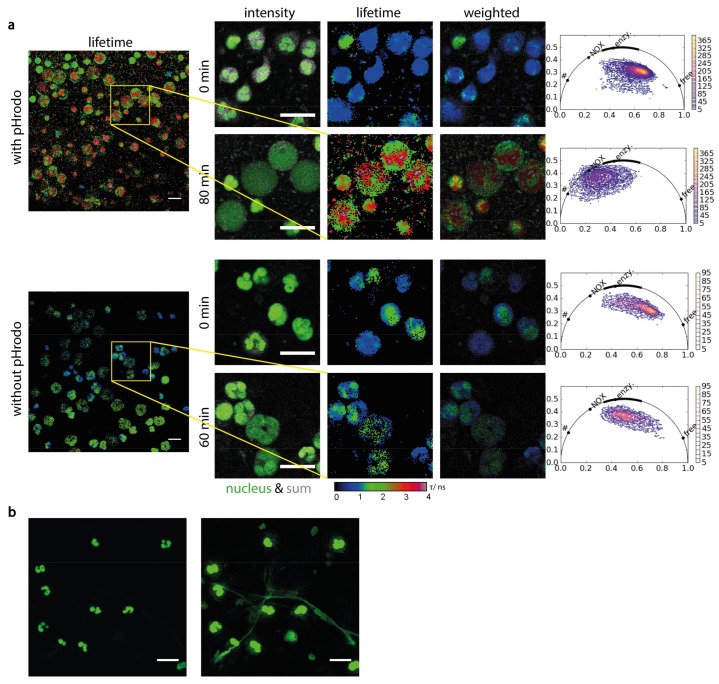
Orchestration of NADPH oxidase activation, cellular death and NET formation (**a**) Comparison of neutrophil metabolism in presence (first two rows) and absence (last two rows) of *Staphylococcus aureus* pHrodo™ beads (*n* = 120 cells per individual and condition, two healthy individuals). The images were picked up at the beginning of the measurement, shortly after the pHrodo™ were added to the cells, and 80 or 60 min later. First column show the sum image (grey) merged with Vybrant DyeCycle Green™ stained DNA. The second and third column show original and intensity-weighted fluorescence lifetime images. Both the color map of FLIM images and phasor plots show the activation of NADPH oxidase in the neutrophils in presence of pHrodo™ beads after 80 min, whereas NETosis can be seen in the Vybrant DyeCycle images. The FLIM images and phasor plots of the other rows remain almost the same, even the nucleus of neutrophils in absence of the beads are slightly swollen. For better visibility, a 124 × 124 pixel area is displayed from the original 512 × 512 pixel images. The phasor plots rely on the total area of 512 × 512 pixels. (**b**) NETs after applying shear forces. DNA was stained by Vybrant DyeCycle Green™, showing in the higher-contrast image the formation of fibrous NETs. Scale bar = 20 µm. In the phasor plots in a “free” encodes free NAD(P)H, “enzym.” NAD(P)H bound to metabolic enzymes, “NOX” NADPH bound to NADPH oxidases and “#” oxidized lipids as defined by Datta et al.

We added Vybrant DyeCycle Green™ to the co-cultures of human PMNs and *S. aureus* pHrodo beads in order to in vivo label the cell nuclei and monitored the process of phagocytosis as previously described. Synchronously acquired fluorescence images of the cell nuclei, of pHrodo beads and NAD(P)H-FLIM maps of PMNs reveal that exploded, misshaped nuclei co-localize with NOX2 over-activation and with phagocytosed beads of low pH, (Figure 5a). The extent of activated NOX (red regions in the NAD(P)H fluorescence lifetime maps) in cells with multi-lobar nuclei, i.e., healthy, intact PMNs, is much lower when compared to dying cells with a large, misshaped nucleus. We could detect phagocytosis but not the early NETosis observed using several vital pathogens, including *S. aureus* [40,41,42].

In contrast, PMNs imaged in the same way after 4 h, when they are naturally dying and without undergoing phagocytosis, do not show a shift in enzymatic activity towards NOX2 activation. In the phasor plot, their signal shifts from the high basic enzymatic activity to the typical signal of free coenzymes, indicating cell death. Also, the nuclei of these cells change from a multi-lobar appearance to a diffuse, round shape in the process of cell death (Figure 5b). The shift in the real component of the phasor plot at the initial time point in PMNs with and without pHrodo and after 60 min, without pHrodo is statistically not significant. In contrast, after 80 min of treatment with pHrodo beads led to a significant increase of the real component in the phasor plot (*p* = 0.002 Student *t*-test) as compared to untreated, dying PMNs (without pHrodo). If shear forces, e.g., through shaking, were applied to the co-cultures of PMNs and *S. aureus* beads, a fibrillary network typical for the process of NETosis was observed (Figure 5b). Hence, the observed misshaped PMN nuclei concretely lead in this situation to NET formation and to immobilization of pathogens.

## 3. Discussion

The use of ultra-short pulsed near-infrared or infrared lasers in intravital multi-photon microscopy boosted our general understanding of highly developed mammalian organisms on a cellular basis in health and disease. Nevertheless, the benefits of the pulsed radiation sources are not only related to the observation of cellular motility and communication but opens new perspectives for the study of cellular and tissue functions, at a molecular level. A prominent example of how cellular and tissue function can be quantified is given by fluorescence lifetime imaging (FLIM). Time-domain FLIM, in general, and time-correlated single photon counting, in particular, require ultra-short pulsed excitation of chromophore molecules followed by a radiation-free time-period during which the fluorescence decay is acquired. The fluorescence decay contains detailed information on the molecular environment exclusively deciding cellular functions and, thus, on the general state of tissues, of organs and of the entire organism. Despite its high potential, up to now FLIM has not reached broad application in biosciences and biomedicine due to technical drawbacks of the complex electronics leading to a poor photon management, especially when endogenous chromophores such as the ubiquitous metabolic co-enzymes NADH and NADPH are monitored. The difficult data interpretation caused by slow, less robust numerical approaches for data evaluation further impeded FLIM application in these research fields. First, latest technological developments allowing the acquisition of high fluorescence signals within the shortest time and model-free evaluation algorithms, as we present in this work, are able to change this paradigm and may enable a broader application for FLIM in life sciences. In order to demonstrate the high potential of our FLIM approach for answering biologically relevant questions, we focused on the phenomenon of phagocytosis in polymorphonuclear cells (neutrophil granulocytes) and monitored it by endogenous NAD(P)H-FLIM over time.

Polymorphonuclear cells (PMNs) are fulfilling their main function, i.e., the clearing of pathogens, especially fungi and bacteria, using various mechanisms. Among these, phagocytosis encompasses pathogen engulfment, followed by activation of NADPH oxidase 2 (NOX2), catalyzing the oxidative burst, and causing complete degradation of the pathogens. In addition, PMNs can undergo NETosis as a pathogen killing process. The molecular mechanisms and dynamics in vivo are not fully understood. Variation in study designs and readout parameters are confusing our understanding of NET formation, for example.

If pathogens outnumber the granulocytes, in order to still ensure their clearing, the cells proceed to programmed cellular death: their nuclear membrane disintegrates, and the leaking DNA forms networks, which can trap the pathogens. This process is known as NETosis. The process of NETosis and its relevance for the clearing of various pathogens have been extensively investigated and discussed. Moreover, a link between NADPH oxidase, phagocytosis and NETosis has been demonstrated by several research groups [43,44].

We present NAD(P)H-FLIM on PMNs isolated from human blood as a unique approach to analyze the association between phagocytically induced NETosis and NOX activation. Furthermore, the NAD(P)H-dependent enzymatic fingerprint spans cellular functions between mere survival and catalysis of massive reactive oxygen species production, aiming to destroy pathogens. Several recent studies showed a direct link between the phenomenon of oxidative burst or oxidative stress and long fluorescence lifetimes detected in cells and tissues. Like Blaker et al., we detected the activation of NADPH-dependent enzymes, i.e., in this context the NADPH oxidases family, to lead to NADPH fluorescence lifetimes of approx. 3600 ps [26,29]. Datta et al. demonstrated that oxidized lipids, as the product of oxidative stress, display an even longer fluorescence lifetime reaching over 7000 ps [37,45]. In contrast to previous studies, which used NOX inhibitors, ROS scavengers or genetic models to address the role of NOX for NETosis induction indirectly, we present here an approach to monitor the enzymatic activity in real time. We found that over-activation of NADPH oxidase, co-localizing with pathogen-containing phagosomes, precedes cell death indicated by high concentrations of free NAD(P)H. Even before cell death, we observed in cells with over-activated NADPH oxidases nuclear membrane disintegration and DNA network formation under the influence of shear forces. Thus, we provided direct evidence that NADPH oxidase activation correlates in time and space with the process of NETosis in PMNs phagocytosing *S. aureus* beads. In contrast, nuclear membrane disintegration and cell death indicated by low or no NAD(P)H-dependent enzymatic activity are not necessarily taking place in a sequential manner but rather arbitrary, following rules that need further investigations. The correlation between NADPH oxidase activation, membrane disintegration and NETosis-related cell death does not hold true for other cell death pathways, since PMNs imaged at late time points after their isolation show high concentration of free NAD(P)H, misshaped cell nuclei, generally indicative for cell death, but no sign of NADPH oxidase activation at any time.

Besides the quite diverse imaging approaches of NETosis in vitro such as imaging flow cytometry or fluorescence microscopy [39,46,47,48], NADPH-FLIM phasor analysis has the advantage not to depend on external labeling as NAD(P)H auto-fluorescence signal is used. Therefore, we believe that this approach has the unique potential to investigate the role of NOX-dependent NETosis also in vivo as we have shown the feasibility of intravital NAD(P)H FLIM in several inflammatory mouse models [3,4,26,27,28].

The protocol presented in this study opens new perspectives for monitoring immune cell metabolism, both in vitro and in vivo. Moreover, we expect that it will facilitate screening molecules having impact on the signaling cascades which trigger NOX-dependent NET formation and, thus, will become a useful tool for designing new therapeutic strategies.

## 4. Materials and Methods

### 4.1. Two-Photon Microscopy Setup for Fluorescence Lifetime Imaging

Experiments were performed using a specialized multi-photon laser-scanning microscope for fluorescence lifetime imaging (FLIM) as displayed in Figure 1a [49]. In brief, the beam of a tuneable fs-pulsed Ti:Sa laser (wavelength range 700–1080 nm, 140 fs, 80 MHz, Cameleon Ultra II, Coherent, Dieburg, Germany) is scanned by two galvanometric mirrors and focused into the sample by an objective lens for deep-tissue imaging (20× dipping lens, NA 1.05, WD 1 mm—Zeiss, Jena, Germany). The resulting fluorescence signal is detected and analyzed either by a TCSPC point detector (FLIM, LaVision Biotech GmbH, Bielefeld, Germany) or photomultiplier tubes (H7422-40, Hamamatsu, Japan). Spectral discrimination of the fluorescence signal was achieved by appropriate dichroic mirrors and interference filters. NADH and NADPH (NAD(P)H) were excited at 760 nm and detected through an interference filter at 460 ± 30 nm. The time step (bin) was 27 ps and the time window for measuring the fluorescence decay was 12 ns. Both pHrodo and Vybrant DyeCycle Green™ (for nuclear staining of live cells) were excited at 760 nm as well and detected at 593 ± 20 nm (pHrodo) and 525 ± 25 nm (Vybrant DyeCycle Green™) respectively simultaneous to NAD(P)H detection.

### 4.2. Neutrophil Granulocytes and CD11b^+^ Monocytes—Isolation from Human Blood

Neutrophil granulocytes (polymorphonuclear cells) were isolated according to published protocols [50]. In summary, granulocytes were isolated from 10 mL of heparin-anticoagulated whole blood with two separate density-gradients under sterile conditions, guarantying minimal pre-activation of the cells of interest by *no touch isolation*. Monocytes were isolated from whole blood as previously described by us [27].

### 4.3. The Phasor Approach to FLIM

In time-domain FLIM, the determination of a fluorescence lifetime is based on fitting the histogram of the photon delay exponentially, while the decay constant is the fluorophore lifetime. Ideally, only a single species of fluorophores populates the observed excitation volume and the temporal decay is mono-exponential. However, most biological samples are highly heterogeneous, which means that their fluorescence decay contains two or even more lifetime components and complicates the analysis, especially if the number of lifetime components is unknown. In that case, the phasor approach [51] is a promising analysis method, since it is model-free. Here the lifetime data are measured in time domain and transferred to a virtual phase domain by numerically calculating the discrete Fourier transform. The sum of all fluorescence lifetimes contained in a pixel is calculated from the normalized real and imaginary result. In case of a mono-exponential decay, plotting those results will give a position on a half-circle termed the phasor (*r* = 0.5, center at (0.5/0)). If the excitation volume contains two fluorescent species, this position in the plot will lie along the straight line connecting the phasors of the pure components. If there are three species, this position would lie within the triangle formed by the line connecting the phasors of the pure components. The phasor analysis was performed using our own routines written in Python and it is available as executable files upon request. Our data analysis was verified by resting and PMA stimulated healthy human monocytes (Figure 2b).

### 4.4. Preparation of the Phagocytosis Assay

After isolation, the PMN cell count was adjusted to 1 × 10^6^ per mL and suspended in RPMI medium without phenol red (Thermo Fisher Scientific, Berlin, Germany) in 12 well plates. Vybrant DyeCycle Green™ nuclear stain (Thermo Fisher Scientific, Berlin, Germany) was added. Directly prior to imaging, pHrodo™ beads conjugated with *Staphylococcus aureus* (Thermo Fisher Scientific, Berlin, Germany) were suspended in 1 mL RPMI medium and introduced drop by drop into the cell suspension, while controlling for a suitable ratio of bacteria to PMNs in bright-field microscopy (typically between 10:1 and 20:1). During the entire imaging experiment, the cell suspension was kept at 37 °C.

## Supplementary Materials

Supplementary materials can be found at http://www.mdpi.com/1422-0067/19/4/1018/s1.
