# Supplementary figures and images for: Phasor-Based Endogenous NAD(P)H Fluorescence Lifetime Imaging Unravels Specific Enzymatic Activity of Neutrophil Granulocytes Preceding NETosis
Source: Int J Mol Sci. 2018 Mar 29;19(4):1018. doi: 10.3390/ijms19041018 (PMC5979388; doi:10.3390/ijms19041018)

only pHrodo in RPMI

intensity

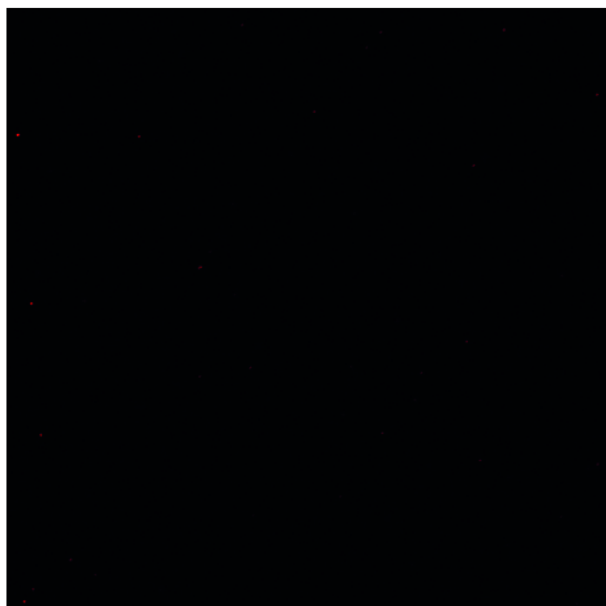

lifetime

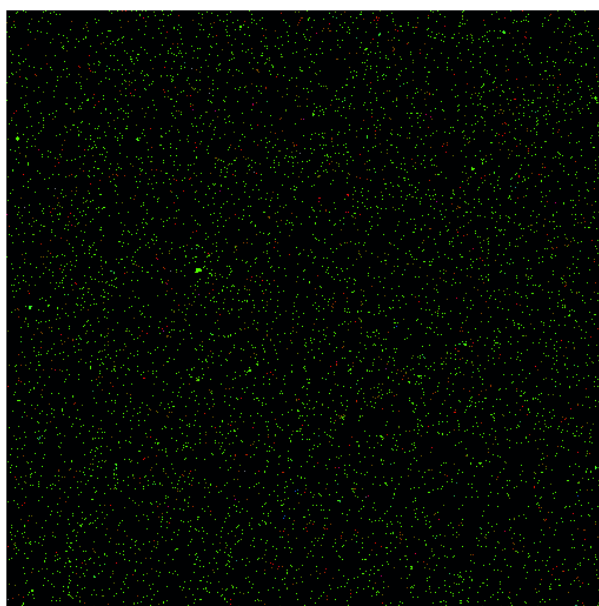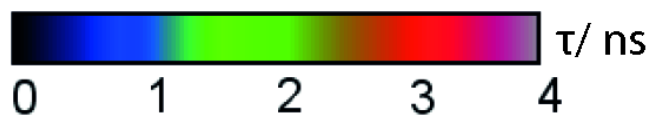

Supplement: Supplementary file 1 [file ijms-19-01018-s001.pdf]
